# Epigenetic Therapy for Solid Tumors: Highlighting the Impact of Tumor Hypoxia

**DOI:** 10.3390/genes6040935

**Published:** 2015-09-25

**Authors:** Shaliny Ramachandran, Jonathan Ient, Eva-Leonne Göttgens, Adam J. Krieg, Ester M. Hammond

**Affiliations:** 1 Cancer Research UK and Medical Research Council Oxford Institute for Radiation Oncology, Department of Oncology, The University of Oxford, Oxford OX3 7DQ, UK; E-Mails: shaliny.ramachandran@oncology.ox.ac.uk (S.R.); jonathan.ient@oncology.ox.ac.uk (J.I.); el.gottgens@gmail.com (E.-L.G.); 2 Department of Obstetrics and Gynecology, University of Kansas Medical Center, Kansas City, KS 66160, USA; E-Mail: akrieg@kumc.edu

**Keywords:** DNA methylation, histone deacetylation, histone methylation, tumor hypoxia, gene-repression, epigenetic drugs

## Abstract

In the last few decades, epigenetics has emerged as an exciting new field in development and disease, with a more recent focus towards cancer. Epigenetics has classically referred to heritable patterns of gene expression, primarily mediated through DNA methylation patterns. More recently, it has come to include the reversible chemical modification of histones and DNA that dictate gene expression patterns. Both the epigenetic up-regulation of oncogenes and downregulation of tumor suppressors have been shown to drive tumor development. Current clinical trials for cancer therapy include pharmacological inhibition of DNA methylation and histone deacetylation, with the aim of reversing these cancer-promoting epigenetic changes. However, the DNA methyltransferase and histone deacetylase inhibitors have met with less than promising results in the treatment of solid tumors. Regions of hypoxia are a common occurrence in solid tumors. Tumor hypoxia is associated with increased aggressiveness and therapy resistance, and importantly, hypoxic tumor cells have a distinct epigenetic profile. In this review, we provide a summary of the recent clinical trials using epigenetic drugs in solid tumors, discuss the hypoxia-induced epigenetic changes and highlight the importance of testing the epigenetic drugs for efficacy against the most aggressive hypoxic fraction of the tumor in future preclinical testing.

## 1. Cancer Epigenetics

The building blocks of chromatin are nucleosomes. Each nucleosome constitutes 146 base-pairs of DNA wound around a histone octamer consisting of histones H2A, H2B, H3 and H4. Nucleosomes are connected by linker DNA and the linker histone H1, extending the length of nucleosomal DNA to approximately 16–180 base-pairs. This “beads on a string” arrangement of nucleosomes is further condensed into secondary and tertiary levels of compaction (the so-called 30 and 100 nanometer fibers), with progressively higher levels of compaction culminating in the familiar mitotic chromosome. This enables the compression of meters of DNA into a single cell while also permitting dynamic changes in chromatin structure, which is necessary to balance the need for cellular packaging with the mobilization-essential functions including transcription, replication and repair [1]. Regions of chromatin can maintain either an open conformation called euchromatin, which is associated with active transcription, or closed conformation known as heterochromatin, which is associated with gene-repression [2]. The maintenance of euchromatin and heterochromatin is dictated by epigenetic mechanisms, which include changes in DNA methylation and histone modifications. Generally, DNA hypermethylation and histone H3K9, H3K27 and H4K20 methylation are associated with gene-repression, which refers to the reversible decrease in gene expression [3,4]. DNA hypermethylation and H3K27me3, however, can also be associated with gene-silencing, which is the long-term inhibition of gene expression [5,6,7]. On the other hand, DNA hypomethylation, methylation of H3K4 and H3K36, and acetylation of H3 and H4, generally mark areas of active gene expression [3,4], although H3K36 methylation can also be associated with methylated CpG islands, a repressive mark [8]. Emerging evidence has established a significant role for epigenetic changes in promoting cancer [3]. Epigenetic regulation may directly affect carcinogenesis [9], metastasis [10], drug resistance [11] and relapse [12]. Ascertaining which epigenetic changes are cancer-associated and how these changes promote cancer, are critical to designing strategies to reverse the cancer-associated epigenetic changes for cancer therapy.

Abnormalities in the epigenome arising from changes in promoter region CpG methylation and histone post-translational modifications can lead to dysregulated gene expression in cancer cells. The epigenetic repression of tumor suppressors, *Breast cancer 1* (*BRCA1*); *PYRIN-PAAD-DAPIN domain* (*PYD*) and *caspase-recruitment domain* (*CARD*) *domain containing* (*PYCARD*) which encodes Apoptosis-associated speck-like protein containing a *CARD* (ASC); and *Suppressor of Cytokine Signaling* (*SOCS*), can greatly contribute to cancer progression [13,14,15]. The repression of these tumor suppressors was attributed to DNA hypermethylation and histone hypoacetylation [13,14,15]. Additionally, candidate tumor suppressors *retinoic acid receptor responder* (*tazarotene induced*) *1* (*RARRES1*), which encodes Tazarotene-induced gene-1 TIG1; *Dab*, *mitogen-responsive phosphoprotein*, *homolog 2* (*Drosophila*) (*DAB2*); *Ras association* (*RalGDS/AF-6*) *domain family member 1* (*RASSF1*); and *FEZ family zinc finger 2* (*FEZF2*) were shown to be down-regulated through a DNA hypermethylation-mediated mechanism in nasopharyngeal carcinoma [16,17,18,19]. The down-regulation of tumor suppressors can lead to the outgrowth of tumor cells. Re-expressing the repressed or silenced tumor suppressors by pharmacologically reversing the cancer-associated epigenetic changes, may induce cancer cell death or sensitize the cancer cells to chemo- or radio-therapy [20], making epigenetic drugs a suitable approach to the treatment of cancer.

## 2. Targeting Cancer Epigenetics

**Figure 1:**
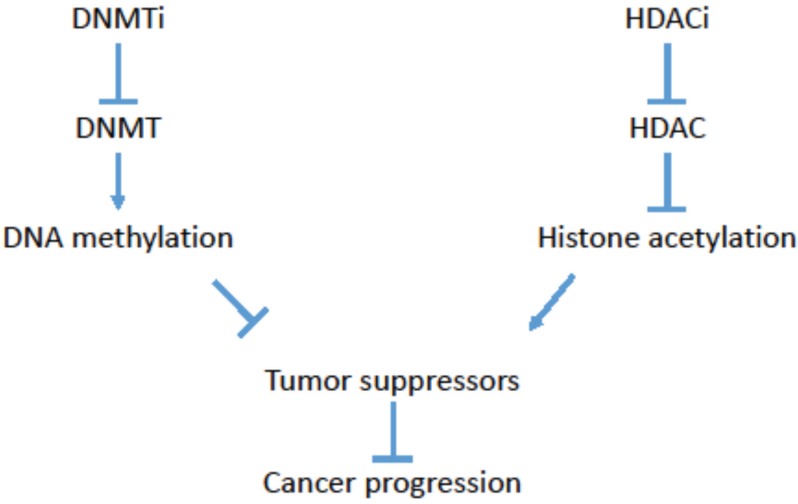
Epigenetic drugs in cancer therapy. A simplified schematic of the effects of DNA methyltransferase inhibitors (DNMTi) and histone deacetylase inhibitors (HDACi) on cancer progression.

As DNA hypermethylation has been linked to cancer progression, clinical studies have focused on inhibitors of DNA methyltransferases (DNMT) as a potential therapeutic approach to reverse this cancer-promoting epigenetic change (Figure 1) [21,22]. DNMTs targeted by these inhibitors include DNMT1, DNMT3a and DNMT3b. DNMT1 functions as the maintenance methyltransferase, recognizing 5-methyl-cytosine on the parent strand during DNA replication and methylating the daughter strand. In contrast, DNMT3a and DNMT3b are considered to be *de novo* methyltransferases, and can establish novel methylation patterns [21]. The DNMT inhibitors tested thus far include 5-Azacytidine and Decitabine. 5-Azacytidine, a nucleoside-analog, incorporates into the DNA during replication and covalently binds to DNMTs, thus reducing the pool of available DNMTs and effectively leading to DNMT inhibition [23]. 5-Azacytidine also has the ability to reverse gene-silencing by affecting histone methylation, for instance, by specifically reducing H3K9me2 and increasing H3K4-methylation at the *p14ARF*/*p16INK4a* locus [24]. Decitabine was subsequently developed as potentially a more potent analog of 5-Azacytidine, given that Decitabine can be more readily incorporated into DNA instead of both DNA and RNA [7]. Decitabine has proven to be more efficacious against the L1210 leukemia cells both *in vitro* and *in vivo* experimental designs [25]. However, the toxicities associated with Decitabine, in particular febrile neutropenia, remains an issue for the use of Decitabine in the clinic [7]. Developing more specific derivatives of the DNMT inhibitors with reduced toxicity would be beneficial for future clinical studies.

DNA methylation is directly linked with histone deacetylation, as DNMT1 has been shown to interact with the histone deacetylase (HDAC) HDAC1 [26,27]. HDAC1 belongs to a larger family of enzymes, which removes the acetylation mediated by histone acetyltrasferases [28]. An interaction between DNMT1 and HDAC1 can result in genes consisting of both hypermethylated DNA and hypoacetylated histones. Akin to DNA hypermethylation, hypoacetylation of histones H3 and H4 have also been linked to cancer progression [13,14,15]. As a result, HDAC inhibitors that result in increased histone acetylation have also been considered as a potential epigenetic therapy in cancer treatment (Figure 1) [21,22]. These HDAC inhibitors were designed to reverse histone deacetylation-mediated repression of tumor suppressors. HDAC inhibitors include hydroxamic acids (Vorinostat, Panobinostat, Belinostat), cyclic tetrapeptides (Romidepsin), short chain fatty acids (Valproic acid), and benzamides (Entinostat) [29].

DNMT and HDAC inhibitors have shown promising results against hematological malignancies. Decitabine has been FDA-approved for acute myeloid leukemia (AML) [30], Vorinostat and Romidepsin have been FDA approved for the treatment of cutaneous T cell lymphoma [31], and Romidepsin and Belinostat have passed FDA approval for peripheral T cell lymphoma [32]. However, it is notable that these epigenetic drugs have met with less success against solid tumors (Table 1). Based on studies in hematological malignancies, it has been suggested that using a lower dosage of the DNMT inhibitors, 5-azacytidine and Decitabine, may prove to be more beneficial in solid tumors [30]. Determining optimal biological dose instead of utilizing the maximum-tolerated dose may lead to reduced toxicity while providing sufficient anti-tumor effects [30]. Combination therapy of certain HDAC inhibitors such as Vorinostat and Belinostat, with chemotherapeutic agents has shown more positive results relative to monotherapy [33,34], and this provides further avenues in therapeutic strategies against solid tumors. Identifying prognostic biomarkers may also prove to be beneficial in selecting appropriate candidates for epigenetic therapy [34]. However, a key difference in hematological malignancies and solid tumors is the abnormal vascularization observed in solid tumors, and the associated solid tumor microenvironment [35]. Understanding the solid tumor microenvironment is pivotal to advancing the use of epigenetic drugs in solid tumor treatment.

**Table 1:** Clinical trials with epigenetic drugs in solid tumors. Summarizing the results of clinical studies using epigenetic drugs against solid tumors. The drug and epigenetic mark targeted along with the clinical phase and outcome of the trial are provided. NSCLC = Non-small cell lung cancer; CR = Complete response; PR = Partial response; SD = Stable Disease.

Drug	Drug Targets	Trials	Combined Therapy	Cancer	Outcome	Reference
Vorinostat	HDAC	Phase II	Monotherapy	relapsed or refractory breast, colorectal NSCLC; metastatic breast cancer; platinum-refractory ovarian or primary peritoneal carcinoma	Toxicities observed, including Grade 3. No responses observed.	[36,37,38,39]
		Phase II	carboplatin and paclitaxel	advanced-stage NSCLC	confirmed response rate of 34% *versus* 12.5% with placebo (*p* = 0.02)	[40]
Romidepsin	HDAC 1 and 2	Phase II	Monotherapy	metastatic renal cell cancer	1 CR and 1 PR in 29 evaluable patients, overall response rate of 7%	[41]
		Phase II	Monotherapy	lung cancer; colorectal cancer; castration-resistant prostate cancer; small cell lung cancer	No objective or minimal responses observed	[42,43,44,45]
Belinostat	HDAC	Phase II		solid tumors	Monotherapy trials not very successful but in combination with chemotherapy (Carboplatin and Paclitaxel) showed benefits	[34]
		Phase II		thymic carcinomas	No objective response	[46]
Panobinostat	HDAC	Phase II		refractory renal carcinoma	No objective response	[47]
		Phase II	Bortezomib	advanced pancreatic cancer	No objective response	[48]
Entinostat	HDAC 1 and 3	Phase II		metastatic melanoma	No objective response	[49]
		Phase II	Erlotinib	advanced NSCLC	No objective response	[50]
		Phase I/II	Azacytidine	metastatic NSCLC	1 CR and 1 PR; 4 of 19 patients had objective responses to future treatments	[51]
Valproic acid	HDAC I and IIa	Phase II	Hydralazine and chemotherapy	various carcinomas	4 PR and 8 SD of 15 patients evaluable for response	[52]
		Phase III	Hydralazine and Cisplatin-topotecan	advanced cervical cancer	Better objective responses observed with combination therapy	[53]
5-Azacytidine	DNMT	Phase I	Erlotinib	solid tumors	Recommended dose/schedule for Phase II	[54]
		Phase Ib–IIa	Monotherapy	epithelial ovarian	1 CR, 3 PR and 10 SD in the 29 evaluable patients	[55]
Decitabine	DNMT	Phase I	Carboplatin	solid tumors	Recommended dose/schedule for Phase II	[56]
		Phase II	Cisplatin	squamous cell carcinoma of cervix	38.1% PR, 23.8% SD; Significant toxicities observed including Grade III and IV neutropenia	[57]

## 3. The Hypoxic Tumor Microenvironment

**Figure 2:**
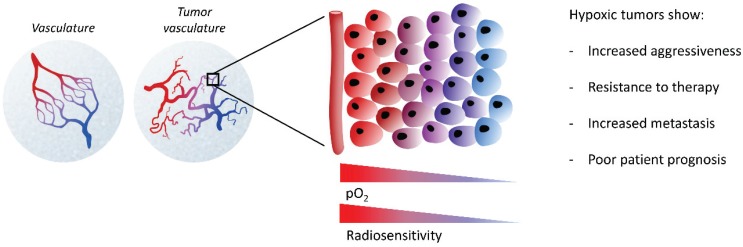
Tumor microenvironment and hypoxia. The majority of solid tumors are characterized by abnormal tumor vasculature. When the high rate of tumor growth cannot be sustained by tumor angiogenesis, this causes limited oxygen supply to the tumor cells distal to the blood vessels, forming regions of hypoxia. Hypoxic tumor cells exhibit increased aggressiveness and metastasis, and are resistant to radiation and chemotherapy.

The microenvironment of solid tumors is characterized by regions of low oxygen (hypoxia), which plays a pivotal role in tumor progression. Tumor hypoxia arises from the high rate of tumor growth that cannot be sustained by a limited oxygen supply (Figure 2). Hypoxia is linked to increased aggressiveness of the tumor, and resistance to all available modalities of cancer treatment, including chemotherapy, radiotherapy and indirectly surgery [35,58,59]. Importantly, numerous studies demonstrate that tumor hypoxia correlates with poor patient prognosis [58,60].

**Figure 3:**
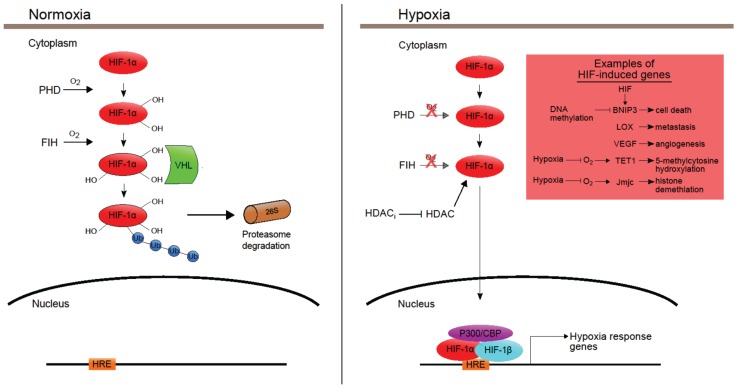
Regulation of HIF family of transcription factors. HIF binds to hypoxia response elements (HRE) to activate transcription of the target genes. The HIF heterodimer is composed of a HIFα subunit and HIF1β subunit. HIFα is oxygen-sensitive and regulated by Prolyl-hydroxylases (PHD) and Factor Inhibiting HIF1 (FIH). In normoxia (at physiological oxygen levels) HIF1α is hydroxylated by PHD proteins leading to Von Hippel Lindau (VHL)-mediated ubiquitination of HIF1α and its subsequent degradation by the proteasome. In normoxia, FIH also mediates hydroxylation of HIF1α, preventing its interaction with p300/CBP, which is required for HIF-mediated transactivation. The oxygen-dependent PHDs and FIH are rendered inactive in hypoxic conditions leading to HIF1α stabilization. HIF1α stability also requires histone deacetylase (HDAC) activity, which can be blocked by HDAC inhibitors that lead to HIF1α destabilization. Under hypoxic stress, HIF1α is stabilized and it interacts with HIF1β and p300/CBP, and the complex binds to HRE to mediate transcriptional activation of hypoxia response genes, including *BNIP3*, LOX, *VEGF*, JmjC demethylases and TET1 hydroxylase. Although *BNIP3* is a HIF target, DNA hypermethylation can block the HIF-mediated transcriptional activation of *BNIP3*. Although some JmjC demethylases and TET1 hydroxylase may be HIF targets, under severe hypoxia, certain members of these dioxygenase proteins may be rendered inactive due to their functional dependency on oxygen.

Hypoxia leads to the induction of the oxygen-sensitive master regulator Hypoxia Inducible Factor (HIF) family of transcription factors, which take control of the cellular response to hypoxia [61]. HIF transcription factors bind to hypoxia response elements (HRE) in target genes to mediate transcriptional activation (Figure 3). The HIF heterodimer is composed of an oxygen-labile HIFα (either HIF1α, HIF2α or HIF3α) and the constitutively expressed HIF1β. In the presence of oxygen, HIFα is hydroxylated by a family of dioxygenases called Prolyl-hydroxylases (PHDs), and this hydroxylated form of HIFα undergoes ubiquitination by the Von Hippel Lindau (VHL) E3 ubiquitin ligase complex, targeting it for proteasomal degradation [62,63]. Additionally, HIF transcriptional activity requires interaction with the transcriptional coactivator p300/CBP among other factors, and this interaction can be blocked by an oxygen-dependent protein called Factor Inhibiting HIF1 (FIH) [64]. In the presence of oxygen, FIH hydroxylates HIF1 and prevents its interaction with p300/CBP, thereby blocking the transcriptional activation of HIF targets [64].

The HIF family of transcription factors regulate many functions, including angiogenesis, metastasis, metabolism, survival and apoptosis [62]. HIF mediates transcriptional activation of a plethora of genes including, *glucose transporter protein type 1* (*GLUT1*), *vascular endothelial growth factor* (*VEGF*), *BCL2/adenovirus E1B 19kDa interacting protein 3* (*BNIP3*) and *lysyl oxidase* (*LOX*) [65,66], which are involved in a variety of cellular functions that promote tumor progression. For instance, *GLUT1* mediates cellular glucose uptake and is important for glycolytic metabolism [66], and *VEGF* plays a key role in angiogenesis and tumor vascularization [67]. HIF-induced LOX expression promoted hypoxia-induced metastasis and has been linked with poor patient prognosis in breast cancer, head and neck cancer and various squamous cell carcinomas [68,69,70,71]. For a detailed understanding of HIF regulation and activity, please see reviews by Nguyen *et al.*, Luo *et al.* and Rankin *et al.* [62,65,66]. Any attempts to specifically target epigenetic mechanisms in hypoxic tumors should account for both the transcriptional and biochemical perturbations imposed by the HIF regulatory axis [62].

## 4. Hypoxia-Induced Epigenetics

Hypoxic tumor cells display a distinct epigenetic profile. Indeed, changes in histone acetylation have been associated with the hypoxic cellular response (Table 2). Hypoxic cells display a decrease in the levels of histone acetylation, and an associated global transcriptional repression [72]. HDAC expression and activity have been shown to be up-regulated in hypoxia, although the mechanism was not investigated [73]. HDAC1 up-regulation was linked to decreased transcription of tumor suppressors *p53* and *VHL* upon exposure to 1% oxygen, and this correlated with increased expression of HIF1α, *VEGF* and angiogenesis [73]. A decrease in histone acetylation on H3K9 in particular, has been associated with down-regulation of genes including tumor suppressors *BRCA1*, *RAD51*, *runt-related transcription factor 3* (*RUNX3*), *p53* and *VHL* in hypoxia [13,73,74,75].

**Table 2:** Epigenetic alterations in hypoxia. Genes that are transcriptionally repressed at hypoxic conditions are regulated by various histone modifications.

Gene	Function	Transcription	Histone Mark altered in hypoxia	Reference
*MLH1*	Tumor suppressor	Repressed	H3K4-demethylation H3K9me2 H3K9-hypoacetylation H3K9me3	[76]
*BRCA1*	Tumor suppressor	Repressed	H3K4-hypomethylation H3K9-hypoacetylation H3K9-methylation	[75]
*RAD51*	Tumor suppressor	Repressed	H3K4-hypomethylation H3K9-hypoacetylation H3K9-methylation	[75]
*RUNX3*	Tumor suppressor	Repressed	H3K9me2 Increased HDAC	[74]
*Tp53*	Tumor suppressor	Repressed	Increased HDAC	[73]
*VHL*	Tumor suppressor	Repressed	Increased HDAC	[73]
*BNIP3*	Tumor suppressor	Repressed	DNA hypermethylation	[77,78]
*APAK*	Negative regulator of p53	Repressed	H3K9me3	[79]
*PP2A-C*	Negative regulator of *ATM*	Repressed	H3K9me3	[80]
*FANCD2*	Fanconi anemia pathway	Repressed	Not Tested	[81]

Along with histone hypoacetylation, DNA hypermethylation may also contribute to gene-silencing under hypoxic conditions (Table 2). Hypermethylation of CpG dinucleotides in the promoter region can block HIF-mediated transcriptional activation of certain targets in hypoxic cells. *BNIP3*, which regulates hypoxia-induced cell death, was found to be repressed by DNA methylation in pancreatic, colorectal and gastric cancer [77,78]. CAIX overexpression has been associated with promoter DNA hypomethylation in gastric cancer, and CAIX expression correlates with tumor advancement and metastasis [82]. Of the PHD proteins, which negatively regulate HIF1α, PHD3 was shown to be regulated by DNA hypermethylation, while PHD1, PHD2 and FIH promoter regions did not display associated methylation changes in hypoxia [83,84].

Contrary to the hypoxia-induced DNA hypermethylation observed at certain loci, hypoxia has been linked to a global reduction in DNA methylation. HIF-dependent methionine adenosyltransferase II, alpha (MAT2A) induction caused reduced levels of *S*-adenosylmethionine, the methyl donor required for DNA methylation, leading to reduced methylation of CpG islands in hepatocellular carcinoma [85]. Hypoxia led to the down-regulation of DNA methyltransferases including DNMT1, DNMT3a and DNMT3b in colorectal cancer cells [86], and a global decrease in DNA methylation [87]. Hypoxia has also been linked with the HIF-dependent up-regulation of the Ten-eleven translocation (TET) dioxygensase TET1, which catalyzes the hydroxylation of 5-methyl-cytosine to 5-hydroxy-methyl-cytosine, in tumorogenic *N*-type neuroblastoma cells exposed to 1% oxygen [88]. TET1 activity essentially leads to DNA demethylation and production of 5-hydroxy-methyl-cytosine, a modification that is associated with active transcription [88]. Hypoxia-induced TET1 up-regulation leading to global DNA hypomethylation, was also demonstrated in scleroderma fibroblasts, in a HIF-independent manner [89]. However, in severely hypoxic conditions, the lack of oxygen may render the oxygen-dependent TET enzymes inactive, and this decreased TET activity may explain the localized hypermethylation observed at specific loci in hypoxia, although this remains to be tested. Although DNA methylation may play a role in gene expression in hypoxia, given that hypoxia is linked to a global decrease in DNMTs and DNA methylation [86,87], it is unlikely to be the predominant epigenetic means of regulating gene expression under conditions of low oxygen.

Recent studies have demonstrated that oxygen levels significantly influence a change in another epigenetic mark, histone methylation, which is distinct from DNA methylation. Hypoxia has been linked to a decrease in the levels of the active histone mark H3K4me3, and an increase in the levels of the repressive marks H3K27me3, H3K9me2 and H3K9me3, at specific genes [72,75,90] (Table 2). Increased levels of the H3K9me2 and H3K9me3 repressive marks, were associated with repression of *mutL homolog 1* (*MLH1*), *BRCA1*, *RAD51*, *RUNX3*, *Ataxia Telangiectasia Mutated* (*ATM*) and *p53-associated KZNF* (*ZNF 420*) *protein* (*APAK*) and *protein phosphatase 2*, *catalytic subunit*, *alpha isozyme* (*PP2A-C*) [74,75,76,80]. Decreased methylation of H3K4 has been associated with repression of *MLH1*, *BRCA1* and *RAD51* in hypoxia [75,76]. Hypoxia has also been associated with a global increase in the methylation of histones H3K4, H3K9, H3K27, H3K36 and H3K79 [72,91,92].

Hypoxia-induced changes in histone methylation may result from changes in the expression and activity of histone methyltransferases. Histone methyltransferases catalyze the methylation of lysine or arginine residues on histones. Lysine residues can be mono-, di- or tri-methylated, and the methyltransferases exhibit high specificity with regards to the substrate, the lysine residue and the extent of methylation [93]. Lysine methyltransferases are categorized based on sequence and structure into two families: (1) suppressor of variegation [Su(var)3-9], enhancer of zeste [E(z)], and trithorax (SET)-domain-containing; and (2) disruptor of telomeric silencing-1 (DOT1)-like [4,93]. G9a, suppressor of variegation 3–9 homolog (Suv39h) 1, Suv39h2 and SET domain, bifurcated 1 (SETDB1) are SET-domain containing methyltransferases that have been implicated in the hypoxic response. Hypoxia can lead to the up-regulation of histone methyltransferase G9a, leading to increased levels of H3K9me2 [74,90]. The Suv39h1 and Suv39h2 methyltransferases remain active in hypoxia leading to increased levels of H3K9me3 [94]. The SETDB1 methyltransferase demonstrated a slight increase in protein levels at <0.1% oxygen, and was found to be important for H3K9me3-mediated repression of *APAK* in hypoxia [79].

On the other hand, hypoxia-induced histone methylation changes may also be affected by histone demethylation. Histone demethylation is carried out by histone demethylases, of which there are two types: Amine-oxidase type Lysine specific demethylases (LSD) and Jumonji C (JmjC)-domain containing oxygenases [95]. Importantly, JmjC dioxygenases require oxygen to function [2,96]. These α-ketoglutarate dependent lysine demethylases have shown oxygen dependence to a certain extent (Table 3) [97], and in 1% oxygen, lack of JmjC demethylase activity can lead to increased histone methylation [92]. *In vitro*, human Jumonji protein JMJD2E demonstrated an almost linear dependence on oxygen, at oxygen concentrations ranging from 0.5%–20.6% [97]. Further, the Km values of JMJD2A, JMJD2C and JMJD2E for oxygen were found to be approximately equal to cellular oxygen concentration, suggesting that these proteins would be sensitive to changes in oxygen levels [98]. Interestingly, α-ketoglutarate showed inhibitory effects on JMJD2C at concentrations above 1 mM, which is similar to the α-ketoglutarate levels found in healthy brain tissue [98]. Gliomas and glioblastomas, on the other hand, have α-ketoglutarate at concentrations of 100–300 µM, and JMJD2C demethylase activity was found to be optimal at ~300 µM *in vitro* suggesting that changes in α-ketoglutarate-levels in cancer cells can also regulate JMJD2C activity [98]. It is also possible that the decreased JmjC demethylase activity in hypoxia may be compensated by increased protein expression [99]. The JmjC demethylases JMJD1A and JMJD2B are targets of HIF transcriptional activation, and were found to be induced at 0.5% oxygen [100]. JMJD2C was also found to be modestly upregulated in hypoxia [100], and at 1% oxygen JMJD2C was shown to interact with HIF1α and promote H3K9me3 demethylation at HREs leading to the optimal transactivation of HIF target genes [101]. HIF-induced JMJD1A expression at 0.5% oxygen caused increased histone demethylation leading to the induction of the *adrenomedullin* (*ADM*) and *growth and differentiation factor 15* (*GDF15*) genes, which promote tumor growth in renal and colon cancer cell lines [102]. Another HIF target, JMJD2B demethylase, was shown to regulate histone methylation of H3K9 at 1% oxygen, promoting tumorigenesis [103]. Although JMJD1A and JMJD2B are both HIF1 targets, when tested in 0.2% oxygen, JMJD2B showed decreased activity while JMJD1A remained active [99], suggesting that some JmjC proteins are more tightly regulated by oxygen than others [104]. Increased levels of H3K4-methylation in hypoxia was attributed to inhibition of Jumonji, AT rich interactive domain 1A (JARID1A) demethylase activity [91]. The H3K4-demethylase Lysine-specific histone demethylase 1A (LSD1) does not rely on oxygen but requires flavin adenine dinucleotide (FAD) to function. LSD1 and PLU-1 (also known as JARID1B) can demethylate H3K4 under hypoxic conditions [76], explaining the decrease in H3K4 methylation observed in certain genes in hypoxia (Table 2). Hypoxic cells display a distinct histone methylation profile due to the significant effects on the oxygen-dependent histone demethylases and possibly due to the activity of histone methyltransferases.

Hypoxia-induced epigenetic changes can lead to the down-regulation of tumor suppressors, providing the hypoxic tumor cells with a selective growth advantage. This is consistent with the finding that hypoxia correlates with increased aggressiveness, metastasis and therapy-resistance of tumors. However, hypoxia-induced histone modifications can also lead to the down-regulation of tumor promoting-genes. Recent work by Olcina *et al.* has described a role for hypoxia-induced H3K9me3 in the repression of *APAK*, a negative regulator of p53 [79]. H3K9me3-dependent repression of *APAK* can lead to the induction of p53-dependent apoptosis, which may have both positive and negative effects on the tumor. p53-dependent apoptosis may play a role in blocking the growth of tumors with functional p53 [79]. However, *APAK*-mediated p53 activation and apoptosis may contribute to the selection of p53-mutant tumor cells [105,106]. This conditional regulation of genes is probably the greatest challenge to developing effective epigenetic therapies. Perhaps with the advent of personalized medicine, specific types of therapies targeting an individual patient’s tumor type and epigenetic spectrum can one day be developed.

**Table 3:** JmjC demethylases in hypoxia. JmjC family of histone demethylases are thought to require oxygen to mediate catalytic function. However, a number of JmjC proteins are induced in hypoxia and targeted by HIF transcriptional activation, or remain active under certain hypoxic conditions. The human JmjC proteins that are known to be induced in hypoxia and activated by HIF are listed below. Additionally summarized are whether each JmjC protein maintains activity in hypoxia.

Human JmjC Proteins	Hypoxia-Inducible [Reference]	HIF Target [Reference]	Activity in Hypoxia [Reference]
KDM2A	Yes [2]		
KDM2B	Yes [2]		
JHDM1D	Yes [2]		
PHF8	Yes [2]		
PHF2	Maybe [2]		
JMJD8			
KDM3A/JMJD1A	Yes [2]	Yes [100,102]	Active at 0.2% oxygen [99]
KDM3B	Yes [2]		
JMJD1C	Yes [2]		
Hairless			
JMJD4			
JMJD6	Yes [2]		
HSPBAP1			
HIFAN	No [2]		
KDM4C/JMJD2C	Yes [2]	Yes [100]	
KDM4A/JMJD2A			
KDM4B/JMJD2B	Yes [2]	Yes [100,102]	Inactive at 0.2% oxygen [99]
KDM4D	Yes [2]		
KDM4E/JMJD2E	-	-	Graded decrease with decreasing levels of oxygen at a range of 0.5%–20.6% oxygen [97]
KDM5D	Yes [2]		
KDM5C	Yes [2]		
KDM5B/JARID1B	Yes [2]	Yes [102]	
KDM5A			
KDM6A	Yes [2]		
UTY			
KDM6B	Yes [2]		
JARID2	Yes [2]		
JMJD7			
JMJD5			

## 5. Effects of Epigenetic Drugs in Hypoxia

Given the distinct profile of the chromatin landscape in hypoxic tumor cells, and given that the hypoxic cells tend be the most aggressive and therapy-resistant, it is essential to understand the effects of epigenetic drugs in hypoxia. Although DNMT inhibitors have undergone Phase I clinical trials in solid tumors, the efficacy of these drugs against the hypoxic fraction has not been validated. While DNA hypermethylation may play a role in silencing some genes in hypoxia, a global down-regulation of DNMTs and DNA methylation has also been observed [86,87], raising the question of efficacy of DNMT inhibitors under hypoxic conditions. Furthermore, Lachance *et al.* demonstrated that DNMT3a mediates silencing of endothelial PAS domain protein 1 (*EPAS1*), which encodes HIF2α, in renal epithelial cells, and that the loss of DNMT3a can lead to the induction of HIF2α, which provides these tumor cells with a selective growth advantage in hypoxia [107]. A switch to the HIF2α hypoxic response can generate tumor stem cell-like properties leading to more aggressive cancers [108]. Given the potential for DNMT3a inhibition to switch towards an aggressive HIF2α hypoxic phenotype, it is important to fully understand the biological consequences of using DNMT inhibitors. This finding highlights the need to test the effects of DNMT inhibitors in hypoxia prior to investigating the efficacy in solid tumor treatment.

The effects of HDAC inhibitors under hypoxic stress have been investigated. While HDAC inhibitors cause histone hyperacetylation, increasing evidence has demonstrated a direct effect on HIF transcription factors in hypoxia, affecting both the expression and function [64]. HDAC4 is known to positively regulate HIF1α under hypoxic stress [109]. Indeed, HDAC inhibitors negatively regulate HIF thereby impeding angiogenesis [63,64]. The HDAC inhibitor Romidepsin (FK228) blocks HIF1α expression and activity in a lung carcinoma model [110], possibly through histone deacetylation, although the mechanism was not tested. Panobinostat, an inhibitor of class I and II HDACs, sensitizes non-small cell lung carcinoma cells to cisplatin [111]. Panobinostat leads to the destabilization of HDACs including HDAC4, which increases open chromatin conformation by increasing the levels of histone acetylation, possibly increasing the sensitivity to cisplatin [111]. Down-regulation of HDAC4 also coincided with increased acetylation and destabilization of HIF1α in hypoxia [111]. In this study, Panobinostat also affected the cells at physiological oxygen levels but the effects were more severe in hypoxia (1% oxygen). Vorinostat has been tested in hepatocellular carcinoma models, in which it was found to affect HIF1α protein translation but not transcription in the presence of hypoxia-mimetic agents [31]. Interestingly, they also observed a decrease in p53 transcript and protein levels upon treatment with Vorinostat [31]. Although HDAC inhibitors negatively regulate HIF and dampen the HIF hypoxic program thereby destabilizing the tumor hypoxic cells, HDAC inhibitors did not have a strong effect against solid tumors in Phase II trials (Table 1). One major hurdle for the use of HDAC inhibitors in solid tumor treatment is the cardiotoxicity observed, and it is possible that this may be overcome by selective delivery of the drug specifically to the region of interest [112]. Additionally, the effects of HDAC inhibitors on the epigenetic profile of the hypoxic tumor cells is not completely known, and investigating the HDAC inhibitor-induced changes in gene expression patterns in hypoxia, may provide additional valuable information.

Histone methylation is significantly impacted by oxygen levels [72,80], primarily due to the reduced activity of certain JmjC histone demethylases (Table 3) which require oxygen to function [2,95]. Consequent increase in histone H3K9-methylation is associated with the repression of a number of genes including tumor suppressors (Table 2). Decreased levels of histone H3K4-methylation has also been linked with silencing of tumor suppressors in hypoxia (Table 2). Although histone methylation may provide a potential means of targeting the hypoxic tumor cells, the biological outcome of this epigenetic mark is still not fully understood, since histone methylation can repress both tumor suppressors and tumor-promoters, such as *APAK*, as described above. It would be informative to test the effects of targeting histone methylation on gene expression in hypoxia. One method of investigating the effects of histone methylation on gene expression is to inhibit the methyltransferases, and a number of histone methyltransferase inhibitors have been developed [113]. The Suv39h1 methyltransferase inhibitor, chaetocin, has shown reduced H3K9-methylation and re-expression of tumor suppressors p15 and E-cadherin in AML cells [114], and in human leukemia cells [115]. The LSD1 demethylase inhibitors biguanide and bisguanidine, which are polyamine analogues, showed promising results in colon carcinoma cells [116,117]. These LSD1 inhibitors lead to the re-expression of *secreted frizzled-related protein* (*SFRP*s) and *GATA* family transcription factors, which were silenced in colon cancers, and this was correlated with an increase in H3K4me2 and H3K9-acetylation, and a decrease in H3K9me1 and H3K9me2 [116,117]. Investigating histone methylation as a potential target for future preclinical studies may be beneficial.

It is known that numerous genes are repressed in hypoxia, either though epigenetically regulated transcriptional repression (Table 2) or due to the reduced enzymatic activity of proteins that are oxygen-dependent. Therefore, when developing a compound for solid tumor therapy, it is important to understand whether this target remains active in the most aggressive part of the solid tumor, the hypoxic regions. For instance, several compounds have been in consideration for JmjC inhibition, including Fe(II) and α-ketoglutarate oxygenase inhibitors such as *N*-oxalyl glycine and its derivatives; and the hydroxamic acid-based HDAC inhibitor Suberoyl Anilide Hydroxamic Acid (SAHA) and its derivatives; and pyridine carboxylates [118]. It is important to note that *N*-oxalyl glycine and some pyridine carboxylates may also inhibit PHD2 and FIH, which may activate the HIF pathway and confound anti-tumor activity [118]. However, the effectiveness of these JmjC inhibitors on the most aggressive, severely hypoxic tumor cells is uncertain given that some JmjC dioxygenases may already be inactive under these conditions. It is imperative that compounds be tested pre-clinically for efficacy against hypoxic tumor cells before moving on to clinical trials in solid tumors.

## 6. Conclusions

There has been an increased focus on epigenetic drugs in cancer therapy. While DNMT and HDAC inhibitors have shown promising results against hematological malignancies, they have proven to be less effective against solid tumors. Most, if not all, solid tumors have regions of hypoxia, and, as discussed above, hypoxic tumor cells display a distinct epigenetic profile. In particular, hypoxic tumor cells have increased levels of the repressive histone methylation marks. We propose that when developing epigenetic drugs against solid tumors, given that the most aggressive hypoxic regions of the tumor display a distinct epigenetic profile, it is important to test the effects of these drugs under hypoxic conditions prior to clinical trials. Furthermore, given the global trend towards transcriptional silencing and the reduced function of oxygen-dependent enzymes, such as JmjC demethylases and TET hydroxylases, it is important to understand the epigenetic changes in hypoxia, and the associated biological consequences in order to design effective epigenetic drugs against solid tumors.
